# Salivaricin G32, a Homolog of the Prototype *Streptococcus pyogenes* Nisin-Like Lantibiotic SA-FF22, Produced by the Commensal Species *Streptococcus salivarius*

**DOI:** 10.1155/2012/738503

**Published:** 2012-04-08

**Authors:** Philip A. Wescombe, Kristin H. Dyet, Karen P. Dierksen, Daniel A. Power, Ralph W. Jack, Jeremy P. Burton, Megan A. Inglis, Anna L. Wescombe, John R. Tagg

**Affiliations:** ^1^BLIS Technologies Ltd., Centre for Innovation, University of Otago, P.O. Box 56, Dunedin 9054, New Zealand; ^2^Department of Microbiology and Immunology, University of Otago, P.O. Box 56, Dunedin 9054, New Zealand

## Abstract

Salivaricin G32, a 2667 Da novel member of the SA-FF22 cluster of lantibiotics, has been purified and characterized from *Streptococcus salivarius* strain G32. The inhibitory peptide differs from the *Streptococcus pyogenes*—produced SA-FF22 in the absence of lysine in position 2. The salivaricin G32 locus was widely distributed in BLIS-producing *S. salivarius*, with 6 (23%) of 26 strains PCR-positive for the structural gene, *slnA*. As for most other lantibiotics produced by *S. salivarius*, the salivaricin G32 locus can be megaplasmid encoded. Another member of the SA-FF22 family was detected in two *Streptococcus dysgalactiae* of bovine origin, an observation supportive of widespread distribution of this lantibiotic within the genus *Streptococcus*. Since the inhibitory spectrum of salivaricin G32 includes *Streptococcus pyogenes*, its production by *S. salivarius*, either as a member of the normal oral microflora or as a commercial probiotic, could serve to enhance protection of the human host against *S. pyogenes* infection.

## 1. Introduction

The *Streptococcus pyogenes* (Lancefield group A streptococcus)-derived streptococcin A-FF22 (SA-FF22) was the first of the streptococcal bacteriocins to be isolated [1] and then characterized both chemically [2, 3] and genetically [4, 5]. Prior to its complete characterization, it was already apparent that SA-FF22 was closely similar to the well-known *Lactococcus lactis* bacteriocin, nisin [1]. Nisin, a widely-used biopreservative agent, is regarded as the prototype of the lantibiotics, a heterogeneous group of lanthionine-containing bacteriocins produced by a wide variety of Gram-positive bacteria. Strain FF22, the producer of the 26-amino acid 2791 Da SA-FF22, gives a characteristic bacteriocin production ([P]-type) inhibitory pattern referred to as 436 when tested against a set of nine standard indicator bacteria in a standardized deferred antagonism bacteriocin-typing protocol [6].

The SA-FF22 genetic locus comprises nine open reading frames arranged in three operons responsible, respectively, for SA-FF22 regulation, biosynthesis, and immunity [7]. Interestingly, immediately downstream of the SA-FF22 structural gene (*scnA*), there is another open reading frame-designated *scnA′* which has close similarity to *scnA*. Although this reading frame has been shown to be transcribed [7], its function has not been established. The inducing factor for upregulation of SA-FF22 production has also not been identified. Many lantibiotics, such as nisin [8] and salivaricin A [9], have been shown to function as the signal peptides for their own upregulation. In the present study we have attempted to identify the signal for the regulation of SA-FF22 transcription and show that this is not the SA-FF22 peptide.

Relatively recently a lantibiotic peptide identical to SA-FF22 was shown to be produced by the food-grade species *Streptococcus macedonicus* and named macedocin [10]. In screening tests of streptococcal strains for their production of bacteriocin-like inhibitory substances (BLISs), we have detected a number of P-type 436 strains in the species *Streptococcus salivarius*, *Streptococcus mutans,* and *Streptococcus dysgalactiae *[11]. In the present study we show that the agent responsible for the P-type 436 BLIS activity of *S. salivarius* G32 is a SA-FF22-like peptide named salivaricin G32.

## 2. Materials and Methods

### 2.1. Bacterial Strains and Culture Conditions

All strains were maintained on Columbia blood agar base (Difco, Sparks, MD) supplemented with 5% human blood and 0.1% wt/vol. CaCO_3_ (BACa) with incubation in 5% CO_2_ in air at 37°C. Liquid culture media were Todd Hewitt broth (THB) (Difco) and THB supplemented with CaCO_3_ (4 *μ*mol·L^−1^) and glucose (0.1% wt/vol.) (THBCaGlu). *S. pyogenes* strain FF22 [4] and *S. salivarius* strain G32 were originally sourced from human throat and human saliva, respectively. *S. dysgalactiae* strains 61 and 67 were from bovine mastitis (courtesy of Dr. B. Jayarao, University of Tennessee). The details of other strains are listed in the tables in which they appear.

### 2.2. Screening for Antimicrobial Activity

BLIS activity was detected using the deferred antagonism method essentially as originally described by Tagg and Bannister [6]. Briefly, a BACa plate was seeded from one side to the other with a 1 cm wide inoculum of the test strain from an 18 hr BACa culture using a cotton swab. Following incubation for 18 hr at 37°C in 5% CO_2_ in air, the 1 cm wide culture growth was removed using a glass slide and the agar surface sterilised by exposure to chloroform vapours for 30 min, followed by airing for a further 30 min. Indicator bacteria (from 18 hr THB cultures) were then inoculated with a swab at right angles across the line of the original diametric streak culture of the test strain and the plate re-incubated for 18 hr at 37°C in 5% CO_2_ in air. Zones of inhibition were scored as − for no inhibition or + if definite interference with the growth of the indicator was evident. Variations to the deferred antagonism method included supplementation of the agar with either 2 mg·mL^−1^ Trypan Blue or 1% MgCl_2_, to test for interference of these agents with BLIS activity. 

### 2.3. Purification of Salivaricin G32

Crude preparations of salivaricin G32 were obtained by freeze-thaw extraction of 80 lawn cultures of *S. salivarius* G32 that had been grown for 18 hr at 37°C in 5% CO_2_ in air on tryptic soy broth (BBL) supplemented with 2% yeast extract (Difco), 1% CaCO_3_, and 0.7% Bacto agar adjusted to pH 6.5 before autoclaving (TsYECa). The freeze thaw extraction process consisted of the agar cultures (entire agar plate including bacterial lawn) being frozen at −70°C and then subsequently thawed. The exudate on thawing was collected and clarified by centrifugation (15300 × g for 25 min). Salivaricin G32 purification from this preparation (volume 2 L) was essentially as described previously for SA-FF22 [3]. The supernatant was passed through an XAD-2 column (bed volume 330 mL Serva, Heidelburg) followed by washing with MQ water, then one bed volume of each of 50% and 70% methanol. The active peptide was eluted in one bed volume of 95% methanol (pH 2). Rotary evaporation removed the methanol and the aqueous phase was clarified by centrifugation at 5000 × g for 15 min prior to loading 500 *μ*L aliquots onto a Brownlee C_8_ reversed phase (RP-300, Aquapore Octyl, 300 Å, 7 U) column. Elution of the inhibitory peptide was achieved in a gradient of 18–30% acetonitrile over 60 min at 1 mL/min pump speed using a Pharmacia FPLC system. The active fractions (detected by spot test assay on a lawn of *Micrococcus luteus* indicator I1) were pooled and further fractionated by HPLC using a reversed phase C_18_ column (Phenomenex Jupiter C_18_, 5 *μ*m, 300 Å, 250 × 4.6 mm) equilibrated in 0.1% trifluoroacetic acid. Elution was in isocratic 45% acetonitrile over 60 min. The active fractions were pooled and analysed by Matrix Assisted Laser Desorption Time of Flight Mass Spectrometry (MALDI-TOF MS). A sample of purified salivaricin G32 was subjected to automated sequential Edman degradation on a gas-phase protein sequencer equipped with an on-line microbore PTH-amino acid analyser (Applied Microsystems).

### 2.4. Cloning and Sequencing of *svnA*

To take advantage of the apparent close similarity of the salivaricin G32 peptide sequence to that of the previously-identified SA-FF22, the SA-FF22 primer pair scnF (5′-GCACCTATCCTTCTGAAGAAAG) and scnR (5′-GCACCTAGGCACATTTTTTCTTCC) was used to amplify the SA-FF22 structural gene, and this was used to probe a series of chromosomal digests of strain G32 (results not shown). A 1.9-Kb *Hin*dIII-*Eco*RI derived restriction fragment containing the salivaricin G32 structural gene (*slnA*) was subsequently cloned into pUC19 using standard techniques [12] and sequenced. The LanM universal primers [13] were used to sequence part of the *slnM* gene in strain G32, and then PCR was used to close the gap between *slnA* and *slnM* in order to obtain the complete sequence of *slnA1* (Figure 3).

### 2.5. Distribution of *scnA* and *slnA* in *S. pyogenes* and *S. salivarius*

The species distribution of the SA-FF22 and salivaricin G32 structural genes was determined using PCR. All amplifications were carried out using Hotmaster taq polymerase (Eppendorf, Hamburg, Germany). Typical PCR reactions consisted of 40.5 *μ*L PCR grade water (Eppendorf), 5 *μ*L of 10 × Buffer (Hotmaster, Eppendorf), 1 *μ*L nucleotide mix (Roche), 1 *μ*L each of the appropriate forward and reverse primers (primer stocks at 0.01 ng·*μ*L^−1^), 0.5 *μ*L Taq (Hotmaster 5 U·*μ*L^−1^), and 1 *μ*L of template DNA. Initial denaturation at 95°C for 2 min was followed by 30 cycles of 95°C for 30 sec, annealing at 60°C for 30 sec and elongation for 30 sec at 65°C. Two percent agarose gels were used to analyse the PCR products.

### 2.6. Megaplasmid DNA Detection Using Pulsed Field Gel Electrophoresis

Using previously described methods [14], pulsed field gel electrophoresis (PFGE) was used to detect the megaplasmid DNA content of *S. salivarius* G32, *S. salivarius* 20P3, *S. dysgalactiae* 61, *S. dysgalactiae* 67, and *S. pyogenes* FF22. One mL of 18 hr THB cultures of the test strains was used to inoculate 20 mL THBs, which in turn were incubated at 37°C in 5% CO_2_ in air to an optical density (650 nm) of 0.5. The cells were then embedded in low-melting-point agarose (SeaPlaque, FMC BioProducts, Rockland, ME, USA) and lysed using 40 mg lysozyme mL^−1^ (Roche), followed by treatment with proteinase K (1 mg·mL^−1^) (Sigma). The proteinase K was removed by six 1 hr TE (10 mM Tris, 1 mM EDTA pH 7.5) washes with shaking (120 rpm) at room temperature. The DNA was separated using a CHEF-DR III Pulsed Field Electrophoresis System (Bio-Rad) over 20 hr in a 1% agarose gel (Pulsed Field Certified Agarose, Bio-Rad Laboratories). An initial pulse time of 6 sec and final of 18 sec was used. The included angle (change in orientation of the field) was 120°, and the gel was run at 4.5 V·cm^−1^ with the buffer maintained at 14°C. Gels were stained with ethidium bromide (5 mg·mL^−1^) and examined by UV transillumination. 

### 2.7. Induction of SA-FF22 and Salivaricin G32 Production

The lantibiotic-producer strain (either *S. pyogenes* FF22 or *S. salivarius* G32) was grown for 18 hr in THB. Putative inducer preparations (freeze thaw extracts of lawn cultures of strains FF22, G32, EB1 (SA-FF22-negative derivative of strain FF22) or strains of various other P-type designations) were added as 20 *μ*L spots on the “test” side of a BACa plate and allowed to dry into the agar. The lantibiotic producer strain was then inoculated (by swabbing from the 18 hr THB culture) over the whole plate and incubated for a specified time. Approximately 1-2 hr before the bacteriocin produced by the uninduced bacteria in the lawn culture was expected to attain inhibitory levels; the bacterial growth was scraped from the agar surface. On the “control” side of the plate, 20 *μ*L drops of inducer extracts equivalent to those previously deposited on the “test side” were added and allowed to dry. The agar surface was then sterilized with chloroform vapour. After airing, an indicator strain culture was inoculated over the entire agar surface and incubated. Zone sizes (diameter in mm) were compared between the test and control halves of the plate and induction was considered to have occurred when, at a particular time, the test zone was at least twice the width of the corresponding control zone. Different times for exposure to the putative inducer preparations were evaluated in each experiment, depending on the anticipated bacteriocin production characteristics of the strain under investigation. 

### 2.8. Genbank Accession Number

The GenBank accession number for the partial salivaricin G32 (*sln*) locus from *S. salivarius* strain G32 is JN831266.

## 3. Results/Discussion

### 3.1. Inhibitory Activity of *S. salivarius* G32

Application of the P-typing scheme allows for a preliminary categorization of bacteriocin-producing strains on the basis of their inhibitory profile against nine standard indicator bacteria [6]. In this test, *S. salivarius* G32 and *S. dysgalactiae* 61 both gave P-type 436 inhibitory profiles identical to that of *S. pyogenes* FF22 (Table 1). This can be considered to be a good preliminary indication that all three strains produce closely-similar inhibitory agents. A further indication of inhibitor similarity was the apparent cross-immunity displayed when each of these producers was tested for sensitivity to the homologous (same strain) and heterologous (other two strains) inhibitory products (Table 1) and also the failure of all three strains to produce detectable inhibitory activity when the P-typing medium was supplemented with either 2 mg·mL^−1^ Trypan blue or 1% MgCl_2_, two agents previously shown to specifically suppress SA-FF22 production by *S. pyogenes* FF22 [22].

### 3.2. Purification of Salivaricin G32

**Figure 1:**
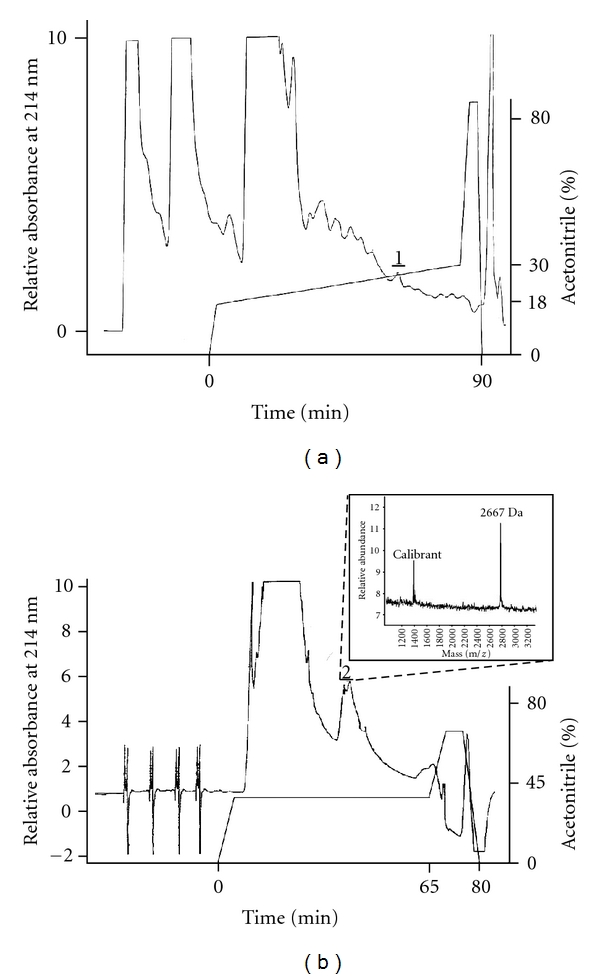
Reversed phase fractionation of salivaricin G32 preparations. (a) Representative C_8_ reversed phase fractionation of concentrated XAD-2 fractions containing inhibitory activity against indicator organism *Micrococcus luteus*. Elution of the column was with a gradient of 18–30% acetonitrile at a flow rate of 1 mL·min^−1^ with detection of absorbance at 214 nm. (b) C_18_ reversed phase fractionation of pooled C_8_ reversed phase fractionated samples having inhibitory activity as indicated in panel A by the solid bar labelled 1. Elution was with isocratic 45% acetonitrile (containing 0.1% TFA) over 60 minutes at a flow rate of 0.7 mL·min^−1^. Active fractions (solid bar 2) from multiple runs were pooled then analysed by MALDI-TOF MS as indicated by the grey insert.

**Figure 2:**
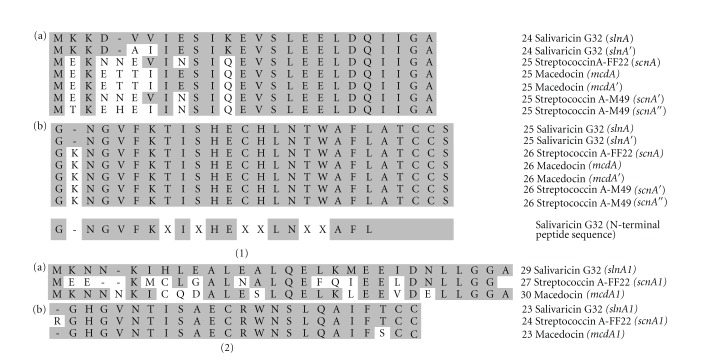
(1) Alignment of (a) the leader sequences and (b) the propeptide sequences of salivaricin G32, SA-FF22, and macedocin (- indicates a spacer introduced to aid the alignment). The N-terminal sequence derived from the purified G32 preparation is shown below, where X indicates a blank cycle in the Edman reaction. (2) Alignment of (a) the predicted translated leader sequences and (b) the predicted translated propeptide sequences for *slnA1*, *scnA1*, and *mcdA1*. The number of amino acids in each sequence is indicated in front of each sequence name.

Salivaricin G32 was purified to apparent homogeneity from TsYECa agar cultures using XAD-2 and a combination of C_8_ and C_18_ reversed phase chromatography (Figure 1). MALDI-TOF MS analysis of the purified fraction identified a mass of 2667 Da (Figure 1), and Edman N-terminal sequencing revealed a partial peptide sequence GNGVFKXIXHEXXLNXXAFL, where X corresponds to an unidentifiable residue or blank cycle. This amino acid sequence corresponds closely to that of SA-FF22, the only difference being the absence of a lysine residue at position two of the salivaricin G32 peptide compared to the SA-FF22 peptide (Figure 2). On the basis of the apparently closely-similar inhibitory spectra of strains G32 and FF22, it appears that this lysine residue difference, although changing the isoelectric point of the molecule, may have relatively minimal impact on the target range of the peptide.

### 3.3. Identification of the Salivaricin G32 Structural Gene

**Figure 3:**
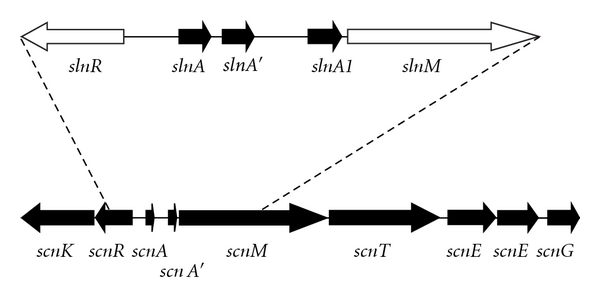
Graphical representation of the SA-FF22 locus illustrating the section of the salivaricin G32 locus that has been sequenced. Black-filled arrows indicate genes that have been completely sequenced. Unfilled arrows indicate genes for which the complete sequence has not yet been determined.

Attempts to amplify the salivaricin G32 structural gene using primers designed to the SA-FF22 locus were unsuccessful and so a cloning approach was taken using the SA-FF22 structural gene *scnA* as a probe for Southern blotting. Following sequencing of the region surrounding *slnA*, the salivaricin G32 structural gene (*slnA*) was shown to be present as two almost identical copies in strain G32 (Figure 3). An alignment of *scnA, mcdA,* and *slnA* and some other known variants is shown in Figure 2. The predicted propeptide resulting from translation of *slnA* differs from SA-FF22 in only the absence of a Lys residue in position 2 of the salivaricin G32 propeptide. However, there are also five different amino acids in the predicted leader sequence encoded by *slnA* and six differences in *slnA′* when compared to the *scnA*-encoded leader region (Figure 2). The first 483 bp of *slnR* was sequenced and the predicted amino acid sequence showed 85% identity to the corresponding region encoded by *scnR*. Similarly, translation of the first 903 bp of *slnM* showed 63% identity to that encoded by *scnM*. Based on these comparisons, it appears that there is significant heterogeneity within the processing genes for the SA-FF22-like lantibiotics indicating a considerable degree of compositional flexibility in a locus that appears to have dispersed widely amongst a variety of streptococcal species.

### 3.4. Distribution of *slnA* and *scnA* in *S. salivarius* and *S. pyogenes*

PCR detected* slnA* in 7 (23%) of 27 BLIS-producing *S. salivarius* (Table 2). In *S. pyogenes,* only thirteen (9%) of 144 tested strains of a wide variety of serotypes were PCR-positive for *scnA* (Table 3). By contrast, 125 of the 144 *S. pyogenes* were determined to be positive for the salivaricin A structural gene (*salA*) (results not shown). Interestingly, *scnA*-like genes were detected in some *S. pyogenes* strains that did not appear to produce SA-FF22-like P-type activity such as the P-type 614 strain M57-71724, the P-type 324 strains M58-78234, M58-71726, M77-79305 and emm109-99454, the P-type 577 strains emm83-60173 and emm113-99458, the P-type 400 strain ST 2037-99448 and the BLIS-negative (P-type 000) strains emm88-60183 and emm105-99449. This indicates that in many *S. pyogenes* the SA-FF22 operon is incomplete, as has previously been shown to be the case in *S. pyogenes* for the streptin and salivaricin A loci [23, 24]. Interestingly, a selection of the *S. pyogenes* positive for *scnA* but apparently not producing SA-FF22 was found to be resistant to SA-FF22 (strains M58-78234, M77-79305, ST 2037-99448, emm105-99449, and emm109-99454), indicating that the immunity component of the SA-FF22 locus was functional for these strains and may provide an ecological advantage in the presence of *S. salivarius* or *S. pyogenes* producing SA-FF22-like inhibitors. In addition, it appears that the salivaricin G32 locus can be expressed as part of a battery of antimicrobial peptides in certain strains such as *S. salivarius* strain T32 [14]. Four *S. pyogenes* strains A1020, FF45, Min19, and Min52 were shown to have two copies of *scnA*, an arrangement similar to that of the *scnA* locus in *S. pyogenes* strain M49 and for the *slnA* locus in *S. salivarius* strains G32 and JH.

These observations indicate that the production of salivaricin G32 could be a useful component of the anti-*S. pyogenes* armoury of *S. salivarius* probiotics. The antibacterial spectrum of the SA-FF22 family of lantibiotics is known to extend beyond streptococci to also include a broad variety of potential pathogens and food spoilage bacteria such as *Listeria*, *Leuconostoc,* and *Enterococcus* [10]. As has been found for nisin-producing strains of the food grade species *Lactococcus lactis, *BLIS-producing *S. salivarius* also has potential efficacy and applicability when added to foods as preservatives, since they are known to be nonpathogenic [25]. Indeed the widely-used BLIS-producing probiotic, *S. salivarius* strain K12, has recently received self-affirmed generally regarded as safe (GRAS) status for addition to food in the USA. One additional potential benefit for the human consumer of food containing salivaricin G32- (or macedocin-) producing bacteria is that the coproduced induction peptide may boost the BLIS activity of salivaricin G32-positive *S. salivarius* present in the consumer's oral microflora, thereby potentially providing increased protection against *S. pyogenes*, as has been previously reported for the stimulation of salivaricin A production by the host's indigenous microflora upon ingestion of inducer peptide-containing milk [26].

### 3.5. Amplification of an *scnA*-Like Gene in *S. dysgalactiae*

A *scnA*-like gene was also detected in *S. dysgalactiae* strain 61 by PCR using primers designed to conserved regions of the SA-FF22 and salivaricin G32 structural genes. The structural gene in *S. dysgalactiae* strain 61 appeared identical to that in strain FF22, although it is possible that the regions defined by these *scnA*-internal primers may differ in strain 61. *S. dysgalactiae* strain 61 was shown to produce a P-type of 436 and freeze-thaw extracts cross-induced production of both SA-FF22 and G32 (Table 4).

### 3.6. Megaplasmid Detection Using Pulsed Field Gel Electrophoresis

**Figure 4:**
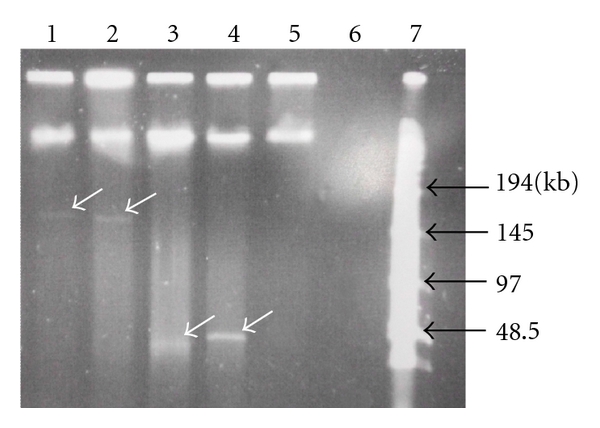
PFGE Analysis of megaplasmid content of total DNA of *S. salivarius* strains G32 (lane 1), 20P3 (lane 2), *S. dysgalactiae* strains 67 (lane 3), and 61 (lane 4), and *S. pyogenes* strain FF22 (lane 5). Lane 6 was left blank and lane 7 contained low range PFG marker (New England Biolabs). Megaplasmid bands are indicated with white arrows and marker sizes are indicated to the right of the gel.

Analysis of *S. salivarius* G32, *S. dysgalactiae* 61, and *S. pyogenes* FF22 showed megaplasmid DNA (ca. 170 kb) to be present only in strain G32 (Figure 4). An apparently intact salivaricin G32 locus has also been detected on a 220 kb megaplasmid in *S. salivarius* strain JH, where it comprises part of a lantibiotic island adjacent to loci encoding salivaricin A and streptin [14] (N. Heng unpublished, 2011). It is interesting to note that *S. pyogenes* strain FF22 does not appear to contain megaplasmid DNA; however, both *S. dysgalactiae* 61 and 67 have similar-sized (48 kb) exogenous DNA fragments (which may be bacteriophage or plasmid). The *S. salivarius* megaplasmids do not therefore in themselves appear to comprise the structural entity whereby lantibiotic determinants translocate to other streptococcal species, but rather, may act as repositories for lantibiotic-encoding DNA acquired from other bacteria.

### 3.7. Induction of SA-FF22 and Salivaricin G32 Production

The bacteriocin production of strains FF22, G32, and 61 was increased (induced) following the addition of freeze thaw extracts from 18 hr TsYECa cultures of either *S. salivarius* G32 or *S. dysgalactiae* 61 and also by 18 hr THB cultures of* S. pyogenes* strain FF22. No enhanced production followed addition of extracts from strains having different P-type profiles or preparations of purified salivaricin G32 (Table 4). The inability of pure salivaricin G32 to induce production of any of this family of SA-FF22 lantibiotics indicates that the signal for upregulation of their production is not the antimicrobial peptide itself, but rather, some other molecule formed by the lantibiotic producer strain. In an attempt to identify the inducing molecule, the SA-FF22-like peptides from a strain FF22 THB culture were separated by XAD-2 chromatography followed by anionic exchange then reversed phase HPLC using a C_18_ column. Individual fractions were assayed for induction of SA-FF22 production in the liquid induction assay. The inducing agent initially copurified with fractions also exhibiting inhibitory activity, but during C_18_ separation it eluted earlier (results not shown). MALDI-TOF MS analysis of inducer active fractions however, only detected peptide having the mass of SA-FF22, indicating that the molecule responsible (even when in very small amounts) for the induction of SA-FF22 production may have copurified with residual (but nevertheless subinhibitory) quantities of SA-FF22 present in that fraction. This is consistent with reports that the amounts of inducer peptide required to induce other lantibiotic systems are also extremely small [8]. Furthermore, the recent report that macedocin is not an autoinducing molecule, but rather that an *α*_S1_-casein medium component can serve to induce macedocin production in laboratory-grown cultures [27], lends further support to our contention that the SA-FF22 family of lantibiotics may not be autoinducible.

## Figures and Tables

**Table 1:** Bacteriocin P-typing of SA-FF22-like strains.

Indicator strain	Description of strain/test	Inhibition of indicator by producer strain*			Indicator strain source/reference
		*S. salivarius * G32	*S. pyogenes* FF22*	*S. dysgalactiae * 61	
*M. luteus *T18	Indicator 1	+	+	+	[6]
*S. pyogenes *FF22	Indicator 2: self/cross test	−	−	−	[6]
*S. constellatus* T29	Indicator 3	−	−	−	[6]
*S. uberis* ATCC 27958	Indicator 4	−	−	−	[6]
*S. pyogenes* 71-679	Indicator 5	+	+	+	[6]
*L. lactis* T-21	Indicator 6	+	+	+	[6]
*S. pyogenes* 71-698	Indicator 7	+	+	+	[6]
*S. pyogenes *W-1	Indicator 8	+	+	+	[6]
*S. dysgalactiae subsp. equisimilis* T148	Indicator 9	−	−	−	[6]
*S. pyogenes *EB1	Cured derivative of FF22	+	+	+	J. R. Tagg*
*S. dysgalactiae *61	Self/cross test	−	−	−	B. Jayarao
*S. salivarius *G32	Self/cross test	−	−	−	J. R. Tagg

*Laboratory collection of J. R. Tagg.

**Table 2:** Distribution of *S. salivarius* lantibiotic structural genes in *S. salivarius* strains of different P-type designations.

*S. salivarius* strain	P-type	Presence of salivaricin gene					Strain source/reference
		*slnA*	*salA*	*sboB*	*sivA*	srtA-like*	
193	777	−	−	−	−	−	[14]
20P3	677	+	+	−	−	−	[15]
220	634	−	−	−	+	−	Laboratory collection of J. R. Tagg
36	777	−	−	−	−	−	[16]
5	677	+	+	−	+	−	[16]
6	777	−	+	+	−	−	[16]
9	676	−	+	−	+	−	[16]
DC135 B	677	+	+	−	+	+	Laboratory collection of J. R. Tagg
DC 156A	636	−	−	−	+	−	Laboratory collection of J. R. Tagg
G32	436	+	−	−	−	+	Laboratory collection of J. R. Tagg
GR	677	−	+	−	−	−	Laboratory collection of J. R. Tagg
H7f	677	+	+	−	−	+	Laboratory collection of J. R. Tagg
H21	777	−	+	+	−	−	[17]
H25	777	−	+	+	−	−	Laboratory collection of J. R. Tagg
JH	677	+	+	−	−	+	[18]
JIM8777	226	−	−	−	−	−	[19]
JIM8780 (CCHSS3)	636	−	−	−	+	−	[20]
JO-1	777	+	−	−	+	−	Laboratory collection of J. R. Tagg
K12	777	−	+	+	−	−	[21]
K30	777	−	+	+	−	−	[17]
K-8P	226	−	−	−	+	−	Laboratory collection of J. R. Tagg
M18	677	−	+	−	+	−	[13]
Min5	777	−	+	+	+	−	[17]
MPS	636	−	+	−	+	+	Laboratory collection of J. R. Tagg
NR	777	−	−	+	−	−	[17]
Pirie	777	−	+	−	−	+	Laboratory collection of J. R. Tagg
Strong SA	777	−	+	+	−	−	[17]

Total positive/total tested		7/27	17/27	8/27	11/27	6/27	

*A lantibiotic structural gene having close homology with the streptin lantibiotic gene *srtA* (O. Hyink, unpublished 2009).

**Table 3:** Distribution of *scnA* in *S. pyogenes* of various M-type/emm-type designations.

*scnA* category	Total	M- or emm-type and strain name (P-type)
Positive for *scnA *	13/144	M57-71724 (614), M57-86152, M58-78234 (324), M58-71726, M77-79305 (324), emm83-60173 (577), emm88-60183 (000), emm97-99440 (777), emm105-99449 (000), emm109-99454 (324), emm113-99458 (577), Trinidad (777), ST 2037-99448 (400),

Negative for *scnA *	131/144	M1-71675 (400), M2-71676 (204), M3-71675 (324), M4-85348 (657), M5-71680 (000), M6-71681 (000), M8-71682 (324), M9-71683 (324), M11-85338 (774), M11-71684, M12-71685 (774), M13-71686 (400), M14-85339 (204), M15-71688 (234), M17-85340 (000), M18-71690 (000), M19-71691 (000), M22-86331 (000), M23-85342 (400), M24-71694 (000), M25-71695 (774), M26-71696 (400), M27-85350 (774), M28-71698 (776), M29-71699 (400), M30-71700 (000), M31-71701 (324), M32-71702 (000), M33-87249 (324), M34-85451 (000), M36-71705 (400), M37-71706 (000), M38-71707, M39-71708 (000), M40-71709 (000), M41-71710 (400), M42-71711, M42-87335 (400), M43-86332 (000), M44-97418 (204), M46-71713, M47-85344 (204), M48-85345 (324), M48-71715, M49-71716 (324), M50-71717 (000), M51-71718 (400), M52-85346 (000), M53-71720 (400), M54-71721 (400), M55-92200, M55-85347 (400), M55-71722, M56-71723 (400), M57-99152, M57-99435, M59-91286 (324), M60-71727 (777), M61-71728 (234), M61-99190, M62-85349 (324), M63-72453 (324), M64-72392, M64-75411 (000), M65-75412 (324), M66-76182 (774), M67-75414 (400), M68-87331 (324), M69-75416 (324), M70-75417 (326), M71-75418 (777), M72-79300 (000), M73-87332 (324), M73-79301, M74-85454 (226), M75-86334 (204), M76-87333 (777), M78-87457 (400), M79-79307 (324), M80-79308 (400), M81-85458 (000), M81-79309, emm76-99436, emm82-60185 (000), emm82-20060182 (324), emm84-60174 (674), emm85-60175 (204), emm86-601176 (657), emm87-60177 (000), emm89-60186 (400), emm89-6182, emm90-60178 (000), emm91-60179 (000), emm92-60180 (000), emm93-60187 (000), emm94-99437 (324), emm95-99438 (000), emm96-99439 (304), emm98-99441 (324), emm99-99442 (654), emm100-99443 (454), emm101-99444 (400), emm102-99445 (400), emm103-99446 (000), emm104-99447 (000), emm106-99450 (000), emm107-99451 (224), emm108-99453 (204), emm110-99455 (726), emm111-99456 (726), emm112-99457 (726), emm114-20000172 (226), emm115-20000173 (204), emm116-20000174 (204), emm117-20000176 (000), emm118-20000177 (226), emm119-20000178 (000), emm120-20000183 (204), emm121-20000185 (000), emm122-20000186 (626), emm123-20000187 (600), emm124-20000184 (204), M28-6-127, M-T14 Alaska788409 (234), M-T14 Alaska78051, M49 Alaska78056 (324), Mneg Alaska78444 (400), stg4545 (Gp G) (000), DK1, JT1 (000)

**Table 4:** Screen of extracts for their potential to induce inhibitor production by members of the SA-FF22 family.

Inhibitor-positive preparation tested for inducing activity	P-type of strain used as the source of inhibitor preparation	Preparation induces inhibitor production in		
		*S. pyogenes* FF22	*S. salivarius* G32	*S. dysgalactiae* 61
*S. pyogenes* FF22	436	Yes	Yes	Yes
*S. pyogenes *EB1	000	No	No	No
*S. salivarius* G32	436	Yes	Yes	Yes
*S. dysgalactiae* 61	436	Yes	Yes	Yes
*L. lactis *C2102	636	No	No	No
*S. mutans *K24	636	No	No	No
*S. mutans* H10	777	No	No	No
*S. mitis* SK653	777	No	No	No
*K. varians* NCC1482	777	No	No	No
Salivaricin G32 (pure)	436	No	No	No
